# Knowledge, Attitude, and Practice (KAP) toward the Novel Coronavirus (COVID-19) Pandemic in a Saudi Population-Based Survey

**DOI:** 10.3390/ijerph18105286

**Published:** 2021-05-16

**Authors:** Abdulrahman H. Alqahtani, Saleh A. Alqahtani, Abdullah S. Alhodaib, Ahmed M. Al-Wathinani, Amin Daoulah, Sameer Alhamid, Salah N. Al-Otaibi, Mohammed Abufayyah, Ahmad M. Wazzan, Saif S. Alshahrani, Yahya S. Almaleh, Abdulmajeed M. Mobrad

**Affiliations:** 1 King Abdulaziz Medical City, Riyadh 14611, Saudi Arabia; qahtani42743@hotmail.com (A.H.A.); awazzan@rpa.gov.sa (A.M.W.); 2 School of Medicine, John Hopkins University, Baltimore, MD 21205, USA; saalqahtani@rpa.gov.sa; 3 King Faisal Specialist Hospital and Research Center, Riyadh 11564, Saudi Arabia; AHODAIB@HOTMAIL.COM (A.S.A.); amindaoulah@yahoo.com (A.D.); dralhamid@gmail.com (S.A.); asalah@kfshrc.edu.sa (S.N.A.-O.); Mabufayyah@rpa.gov.sa (M.A.); ssalshahrani@rpa.gov.sa (S.S.A.); yalmalh@rpa.gov.sa (Y.S.A.); 4 Department of Emergency Medical Services, Prince Sultan Bin Abdulaziz College Emergency Medical Services, King Saud University, Riyadh 11451, Saudi Arabia; ahmalotaibi@KSU.EDU.SA

**Keywords:** COVID-19, knowledge, attitude, practices, Saudi population

## Abstract

Background: The Coronavirus Disease 2019 (COVID-19) outbreak has affected all regions and countries with varying impacts based on infection rates and the associated fatalities. This study aimed to assess knowledge, attitude, and practices (KAP) toward the COVID-19 pandemic among Saudi Arabians. Methods: The study utilized a cross-sectional research design. Web-based questionnaires’ link was sent via emails and social media and sample was 5483 respondents. Purposive sampling ensured only those participants that met the inclusion criteria. Validity and reliability were checked. Results: Most respondents, 67.9%, were aged between 18 and 35 years and highest level of education university. The findings based on the study objectives indicated a high level of knowledge about COVID-19, which indicated early detection can improve treatment by 4701 (85.7%), the disease can be treated at home 84.6%, the disease can be prevented and avoided when precautions are taken 96.8%. Moreover, 37.2% of the respondents still used herbal products to prevent and treat the disease, and 72.1% indicating immediate visit the physician when there are symptoms. Conclusion: Promoting public knowledge about COVID-19 by the Ministry of Health is paramount in defeating this disease. Providing more education and awareness for public to comply with WHO’s recommendation is recommended.

## 1. Introduction

The Coronavirus Disease 2019 (COVID-19) is a fast-spreading disease that has affected all countries with varying impacts on infection rates and fatalities. The international presence within a short period is attributed to globalization through trade and diplomatic relations [1,2,3]. Developed countries, including the United States, have been adversely affected where COVID-19 has grown to become the leading cause of death, surpassing cardiovascular diseases and cancer. COVID-19 is known for its fast-spreading and can be contracted by direct contact with an infected individual. At this stage, the characteristics of the disease are changing [4]. Often, this could be one of the possible ways that the transmission is taking place. The World Health Organization (WHO) declared a global health emergency of international importance and urged all countries to work cooperatively to deter the disease’s rapid dissemination [4]. COVID-19 was later deemed a “world pandemic” by the WHO.

The Middle East reported the first COVID-19 case in late February in Iran, but it had spread to 22 countries in the region by May 6. The region had 224,071 reported cases with 8378 deaths, representing a 3.7% fatality rate [5]. The most affected group are those aged between 15 and 75 years with a median age of 42 years. The primary underlying comorbidities that increase the chances of experiencing severe symptoms are diabetes in men and cardiovascular diseases among women [5]. The disease spreads among people who come into contact with infected surfaces. For instance, droplets from an infected person’s cough, sneeze, and heavy breathing contain the virus [5]. One can get infected by touching contaminated surfaces and wiping their face using their hands without washing with soap and water or a sanitizer [6]. The spread signifies that hygiene, social distancing, and wearing facemasks are the ideal prevention measures for the disease.

The cases in Saudi Arabia are slightly different from those in the region, considering the mean age is 36 years where men are the most affected. About 9.3% of the COVID-19 cases in the country are asymptomatic, signifying a large proportion of affected individuals were presented with symptoms. The median incubation period is 6 days after which the infected case exhibits symptoms such as coughs, fever, and sore throat [7]. About 20.1% of infected cases have underlying conditions, including hypertension and diabetes. The primary symptoms are headache, difficulty breathing, and increased heart and respiratory rates, and upon admission, 4.7% require ICU treatment [7]. Currently, Saudi Arabia continues to implement containment measures to prevent a rise in cases. The Saudi Ministry of Health (MOH) has outlined detailed steps to deter and reduce COVID-19 spread, including city lockdowns, local and foreign travel bans, physical distancing, the use of disposable medical or cloth masks, hand washing, and gathering size restrictions. The COVID-19 pandemic is a new global disaster that has wreaked havoc on all populations around the planet, resulting in a massive number of cases and fatalities in a brief amount of time [8].

The level of COVID-19 awareness influences the attitude and practices people employ to control the condition. Therefore, awareness determines management effectiveness based on the level of adherence to the control measures relevant organizations have provided [9,10]. Health sector stakeholders have taken the leading role in creating awareness regarding the disease, how it spreads, and containment measures to improve management [11,12]. The current containment measures are at country and global levels where the government has imposed restrictions based on the severity levels [13]. The present study aims to assess knowledge, attitude, and practices (KAP) among the Saudi population towards COVID-19 prevention.

## 2. Methodology

### 2.1. Study Design and Setting

This study utilized a cross-sectional survey design to guide the study procedure, sampling and data collection methods, and analysis. Data was collected in November 2020. The COVID-19 situation in Saudi Arabia during this particular time was with 347,282 confirmed cases and the number of recoveries were 333,842, while deaths were 5402. In the other hand the number of hospitalizations were 8038 with 776 in critical conditions. Finally, the total number of PCR Tests were 8,052,694 [14].

The approach entailed gathering data and drawing inferences at a particular point in time [15]. In such a case the researchers conducted the study in Saudi Arabia, targeting the Saudi population living in north, south, east, west, and central regions. The researchers chose the group of Saudi population who have access to internet which are 95.7% of Saudi population [16].

### 2.2. Study Population and Sample

The target population for the study comprised Saudi citizens. The inclusion criteria entailed people aged above 18 years and willing to participate in the study. Foreigners in the country were excluded from the study to get the net perspective regarding effectiveness of the efforts in reducing COVID-19 spread. Conversely, the researchers utilized a purposive technique where only those respondents satisfying the inclusion standards, participated in the study.

### 2.3. Data Collection Tool

A web-based questionnaire was the primary research instrument. The instrument was adapted from a previous study and modified to suit the objectives of this study [17]. The previous study focused on KAP regarding the effectiveness of Cholera vaccination campaigns [17]. The questionnaire comprised four sections where the first included demographic data with four items. The second section concentrated on knowledge; third on attitude, and fourth on practices, each with 17, 11, and 10 items, respectively. Literature review was the primary source of secondary data since the researcher compared the primary data findings with those of previous scholars on a similar topic. The questionnaire was both in Arabic and English to suit the language preferences among the respondents.

### 2.4. Response Rate

The survey targeted a sample of 10,000 people, to which 5483 responded. Therefore, the response rate based on this sample size was 54.83% (5483). This contributed to a low error margin. This rate is enough to give the researcher information for the researcher with a minimal error.

### 2.5. Study Procedure

The researcher sent the surveys on social media and online to broaden the respondent pool. To avoid data from reaching the Population outside the Kingdom, the researcher used the first page of questionnaire to confirm that participant living in Saudi Arabia by choosing the option of living in Saudi Arabia in order to move to the questionnaire’s questions. The questionnaire comprised a consent form, which the respondents were expected to read and accept or reject to participate in the study. On average, the respondents took 15 min to fill the online questionnaire. It had a ‘continue later’ option to enable the participant to resume the survey during periods when they were less preoccupied. The Survey Monkey website compiled the data making it easier to download in word and excel formats. Incomplete questionnaires were discarded during the data cleaning process to increase the internal consistency of the data.

### 2.6. Data Management and Analysis

The study comprised independent and dependent variables. The independent factors included knowledge, attitude, and practices. Conversely, the dependent variables were effectiveness in containing COVID-19 spread. The researcher applied descriptive statistics in characterizing the respondents, and to assess the knowledge levels and attitude towards the COVID-19 containment measures. The study utilized a similar analysis method to identify the practices used to reduce community transmission of the disease. The researcher employed elimination questions to ensure that only individuals of legal adult age participated in the study [18]. The survey data collection website automatically compiled the data, easing the recording process to pave the way for subsequent research activities. The researcher entered the data into the Statistical Package for Social Sciences (SPSS) program version 22 by IBM, Armonk, New York, United States for descriptive analysis. The primary methods included frequencies and percentages. Presentation of the results was in tables, charts, and graphs to ease interpretation and facilitate drawing of conclusions regarding knowledge, attitude, and practices towards the COVID-19 pandemic among the Saudi Population.

### 2.7. Ethical Considerations

The researchers obtained ethics approvals from the Saudi Arabian Research Ethics Committee (REC). The respondents were informed about the purpose of the study using information sheets written in English and Arabic. In each case, signing the consent form was a prerequisite that affirmed their participation in the study based on informed consent. The respondents were also informed that their participation was voluntary, and they could stop their participation at any stage in case they felt any question was infringing on their privacy. The responses were anonymized to keep all the information confidential.

## 3. Results

### 3.1. Demographic Findings

Table 1 illustrates the demographic characteristics of participants. The research aimed to determine the age distribution of the respondents based on four quantifications: 18–35, 36–50, 51–65, and above 65 years. The findings indicate that 67.9% of participants were aged between 18 and 35 years. Males comprised most of the respondents, 59.8%, while females represented 40.2%. This study’s results further show a similar distribution as the education’s level of the entire Saudi participants is 68% as Saudi college-educated, and 80.5% of participants is non-health professionals, and 19.5% health professionals.

### 3.2. Level of Knowledge

**Figure 1:**
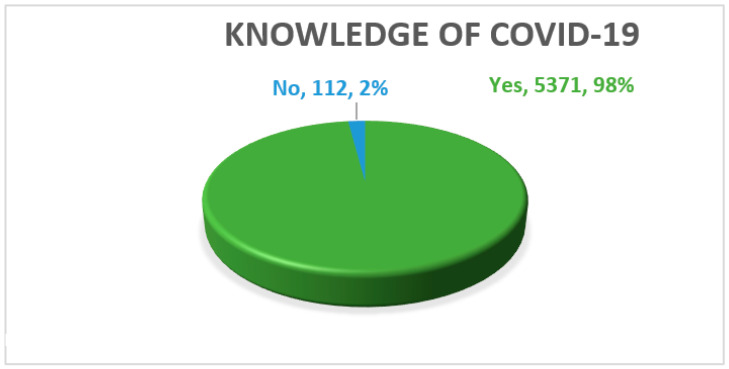
Knowledge of the COVID-19 pandemic. (*n* = 5483).

Figure 1 illustrates the study findings in terms of level of knowledge. The results show that most of the respondents, 5371 (98%), knew about COVID-19 as an infectious disease. However, 112 (2%) of the respondents stated that they were not aware of that.

In addition to knowledge, this study sought to assess the levels of awareness about the pandemic depending on the levels of understanding regarding the sources of information, incubation period, symptoms, treatment, the most susceptible segment of the population, mode of spread, and prevention measures.

Table 2 below summarizes the findings of these objectives. The results indicate the primary source of information about COVID-19 among the Saudi population was social media, 76.9%, followed by television, 19.1%. The other sources utilized on a limited scale were friends’ chat 2.3% and newspapers 2.2%.

Most respondents, 88.5%, knew that the incubation period for COVID-19 was 6–14 days, followed by 6.6% who stated that it lasted between 2 and 5 days. Moreover, 0.8% of respondents stated the incubation period was less than 2 days. A small proportion of the respondents, 4.1%, indicated that they did not know the disease’s incubation period.

The sampled respondents stated that individuals most susceptible to COVID-19 were aged above 50 years, in 92.4% of respondents, followed by those between 31 and 50 years in 4.3%. The least selected were those between 16 and 30 years since they were identified by 1.2% of the total sample.

Most respondents, 30%, were aware that vitamins helped in COVID-19 treatment. They were followed by 28.6% who did not know whether COVID-19 was treatable, and the type of medications applied. Respondents who supported antibiotics were the least represented since they comprised 18.0% of the total sampled respondents.

Most respondents were aware that the hygiene practices were effective in containing COVID-19 spread involving washing hands with soap and water as they comprised 87.4% of the sample. Additionally, 12.3% were aware that the use of hand sanitizer was effective in limiting the spread. Few respondents, 0.4%, considered that the primary hygiene practice was using water only. 

Regarding the causes, most respondents stated that COVID-19 was the outcome of viruses 3282 (59.9%). They were followed by 1434 (26.2%), who attributed the contagious disease to immune deficiency. Additionally, 695 (12.7%) thought that crowding of people caused the emergence of the illness followed by 72 (1.2%), who attributed the contagiousness of the disease to fungi.

Most respondents, 67%, identified social distancing as the ideal COVID-19 prevention strategy followed by wearing facemasks at 26.9%. In such cases, the least represented group suggested wearing gloves and gowns at 0.7% and 5.4%, respectively.

**Figure 2:**
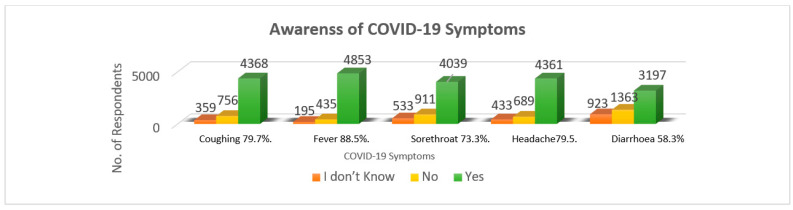
Awareness of COVID-19 symptoms (*n* = 5483).

Figure 2 summarizes the awareness of COVID-19 symptoms. Most respondents identified coughing, fever, sore throat, headache, diarrhea at 4368 (79.7%), 4853 (88.5%), 4039 (73.3%), 4361 (79.5%), and 3197 (58.3%), respectively, as the COVID-19 symptoms.

**Figure 3:**
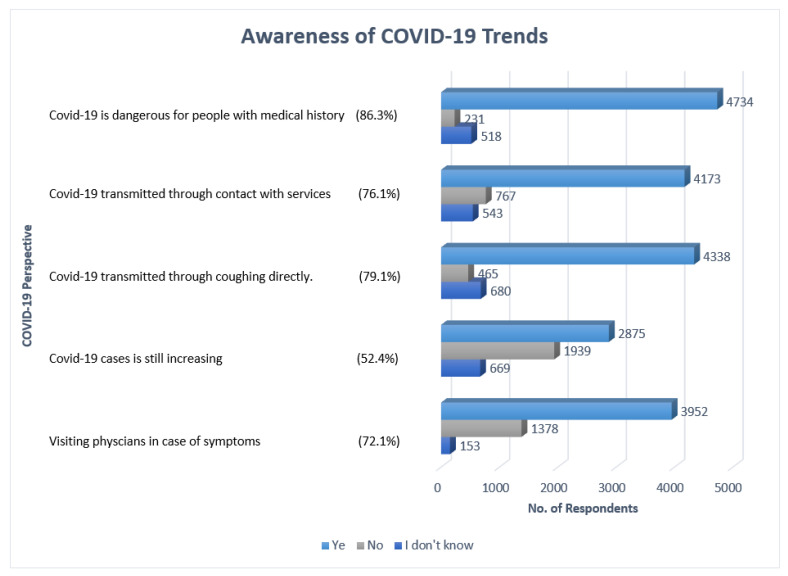
Awareness of COVID-19 trends (*n* = 5483).

Figure 3 summarizes the awareness of COVID-19 trends. Most respondents stated that they were aware of the need to visit the physician in case of exposure, COVID-19 cases are still rising, the disease can be transmitted through coughing directly, and individuals with underlying medical conditions were at the highest risk at 3952 (72.1%), 2875 (52.4%), 4338 (79.1%), and 4734 (86.3%). Additionally, 4173 (76.1%) agreed that contact with infected surfaces increased the chance of contracting the disease.

**Figure 4:**
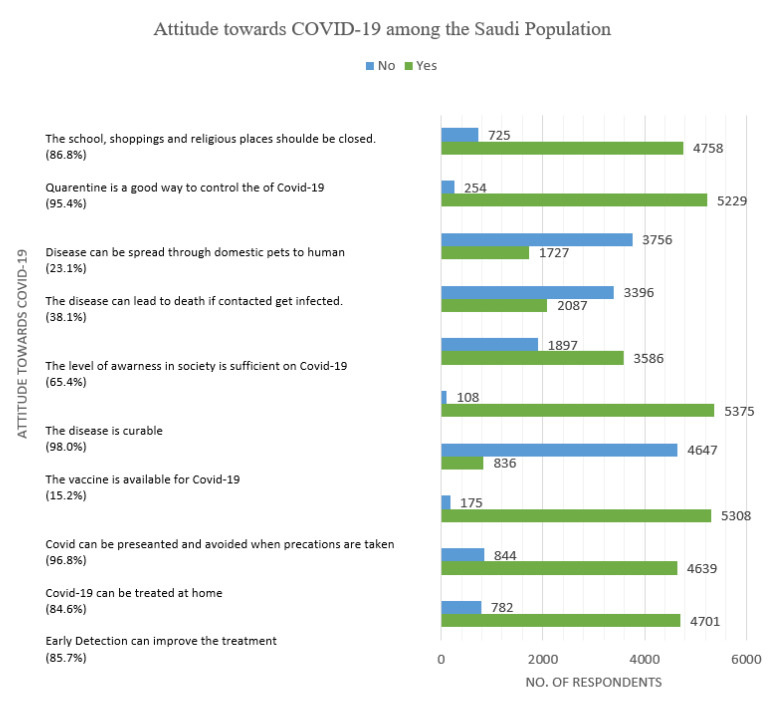
Attitude of the Saudi population towards COVID-19 pandemic (*n* = 5483).

Figure 4 below summarizes the attitudes towards the COVID-19 pandemic. Most respondents agreed to the highlighted factors to be right about the COVID-19 pandemic. For instance, *early detection can improve treatment by* 4701 (85.7%), *the disease can be treated at home at* 4639 (84.6%), *the disease can be prevented and avoided when precautions are taken* 5308 (96.8%), and *the condition is curable* 5375 (98.0%). The majority agreed that *the level of awareness in society is sufficient on COVID-19* 3586 (65.4%), *quarantine is a right way to control the spread of the disease* 5229 (95.4%), and *schools, shopping, and religious places should be closed* 4758 (86.8%).

Nevertheless, most of the respondents were aware about a vaccine for not being available for the disease and developed soon at 4647 (84.8%). The infection leads to death if contacted get infected at 2087 (38.1%) and the disease can be transmitted through domestic pets to humans 1727 (23.1%). The results show the participants had varying perspectives about COVID-19.

### 3.3. Practices to Control the COVID-19 Pandemic

**Figure 5:**
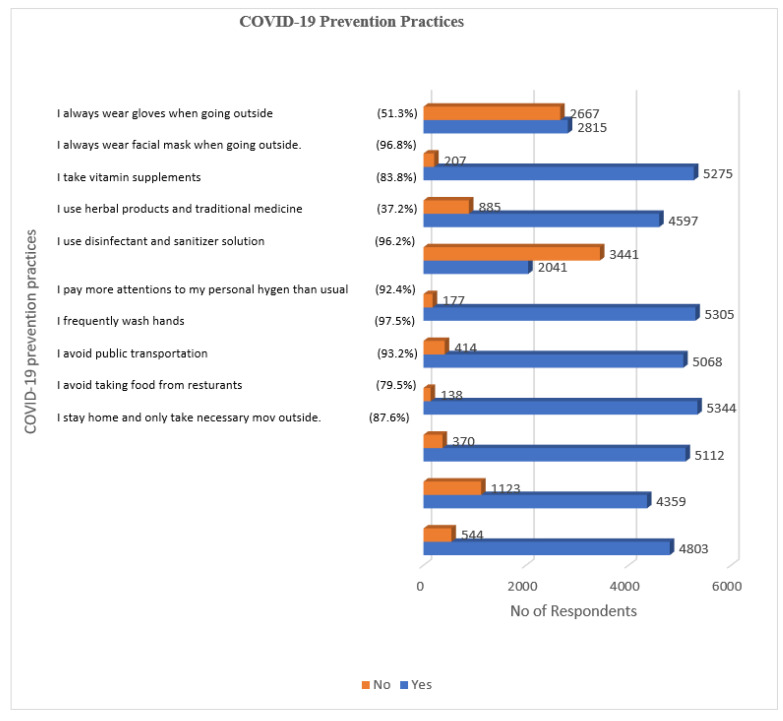
COVID-19 prevention practices by the Saudi Population (*n* = 5483).

The study also aimed to identify the ways the Saudi population has adopted COVID-19 management. Figure 5 below illustrates the results. Most respondents agreed that they *stay at home and only take necessary trips outside* 4803 (87.6%), *avoiding taking food from the restaurants* 4359 (79.5%), and *avoiding public transportation* 5112 (93.2%). In addition, most stated that they *frequently wash hands* 5344 (97.5%), *pay more attention to personal hygiene than usual* 5068 (92.4%), *use disinfectant and sanitizer solution* 5305 (96.2%), *take vitamin supplements* 4597 (83.8%), and *always wear a facial mask when going outside* 5275 (96.8%. About 2815 (51.3%) stated that they *always wear gloves when going outside*. However, more than third of participants undertaken practices were *using herbal products and traditional medicine*, 2041 (37.2%).

## 4. Discussion

Knowledge about the COVID-19 Pandemic, a large segment of the population in Saudi Arabia is aware of the COVID-19 pandemic. This could be attributed to the awareness creation efforts by health sector stakeholders [19]. In addition, the Saudi population could have gotten the information from social media since that is their primary source of news about the pandemic. Saudi Arabia has the region’s highest number of social media users, affirming its significance as the leading source of information [20]. The respondents cited individuals aged above 50 years are the most vulnerable while washing hands with water and soap were the preferred control measures. These findings coincide with WHO recommendations and identified the elderly to have weaker immune defenses against the disease [21]. These results are encouraging, because knowledge of the COVID-19 pandemic is crucial for implementing containment measures since it reduces resistance to change.

In addition to understanding the vulnerable populations and control measures, the respondents were also aware of the COVID-19 incubation period, the causes, prevention measures, symptoms, and the disease trends based on increased cases and what to do in case of suspected exposure. The public health stakeholders in the country are conducting extensive awareness programs to strengthen the population’s responsiveness to government interventions. For instance, the respondents understood that cough, headache, fever, and sore throat were mild COVID-19 symptoms. Additionally, they knew how it spreads and the vulnerability of persons with a medical history to adverse effects of the condition [22]. The results signify that public sensitization exercises in the country have effectively strengthened public vigilance, helping to contain the spread of the disease. In addition, they affirm a high level of awareness since the citizens have information regarding most aspects of the novel coronavirus pandemic from the government and other sources.

The attitude towards COVID-19, how the Saudi population perceived the COVID-19 pandemic was investigated based on the causes, prevention measures, and available treatment options. For instance, the respondents rejected the assumption that domestic pets could spread the virus to humans, and that the disease always ended in death. This attitude coincides with study findings that the condition does not spread from domestic animals to humans. However, the results show that many respondents stated that there were no vaccines for COVID-19 [23]. The results for this point could be attributed to a vaccine having not been approved at the time of the study. Knowledge of the misconceptions about COVID-19 is essential in strengthening adherence to the containment measures.

The respondents supported the government initiative to contain COVID-19 spread by adhering to some of the measures, including the closure of schools and places that involve people gathering and quarantine as control measures. The interventions coincide with the WHO recommendations to contain the spread [24]. Additionally, it was understood that treatment of the disease at home was possible, particularly among asymptomatic patients, as it entails self-isolation to avoid transmitting the disease to other householder members. The response was optimistic that COVID-19 is curable based on the high number of recoveries the country was recording. As such, these findings affirm that some patients recover from the illness without treatment [23,24]. The attitude towards these measures could be attributed to the aggressive awareness creation efforts by public health stakeholders in Saudi Arabia to contain the spread.

Practices Adopted to Control COVID-19, this study investigated the measures the Saudi population has adopted to contain COVID-19 in the country. The results show that the most common practices employed include staying at home and traveling only when necessary, avoiding public places such as restaurants, and regularly washing hands with soap and water or sanitizer. In these terms, the practices coincide with WHO recommendations to reduce community spread in the population [25]. Crowding facilitates the spread of the COVID-19 virus due to limited air circulation and the high likelihood of an infected person being in the group. Therefore, avoiding unnecessary traveling in public areas can reduce the spread since it reduces the chances of contact with an infected person [26]. The practices the Saudi population has employed are effective in managing COVID-19 by decreasing spread.

The respondents identified other practices that are not effective in managing COVID-19; hence they did not engage in them. For instance, most participants disagreed that using herbal products and traditional medicine was effective since the disease does not currently have a known treatment option [27]. The Saudi population appears to understand the finding; hence, they have opted for the practices ‘reliable’ health organizations have recommended. Similarly, the majority of respondents, 62.5%, stated that they do not use herbal products and traditional medicines to prevent or in an attempt to cure the disease. The results signify a small segment of the population still believes in the effectiveness of traditional medicine in managing contemporary conditions. The Saudi population has integrated several practices to control the COVID-19 pandemic spread in the country, highlighting the positive attitude towards containment measures.

### Limitations

The study utilized an online questionnaire, so the entire Saudi population did not have an equal chance to participate in this study since about 5% of population do not have access to the internet [28]. Therefore, the results may not be generalized to whole Saudi population. Furthermore, this study was a cross sectional study undertaken at one point in time and opinions may change. For instance, there were no approved vaccines at the time of the study.

## 5. Conclusions

There is a high level of COVID-19 knowledge in Saudi population with resources and college education. Saudis are aware the disease exists and understand other aspects, including the incubation period, vulnerable members of the population, and the increased risk of contracting the disease among individuals with a history of chronic illnesses. This could be attributed to public sensitization efforts the government has undertaken to control the disease, focusing on the risk factors and measures to reduce exposure to the contagious disease.

The sensitization efforts have also contributed to the positive attitude of the population towards the containment measures since most of the respondents supported the government’s initiatives. For instance, they supported the closure of public places such as shopping centers and schools to limit community spread. This attitude is crucial in promoting public health wellbeing, considering public acceptance of change is a significant milestone in managing contagious diseases.

The effectiveness of the interventions is further evident with the adoption of WHO-recommended practices to contain COVID-19 spread in the population. However, other portion of participants continue to engage in unverified practices to control diseases, including herbal products and traditional medicine for COVID-19 prevention and treatment.

The Ministry of Health (MOH) and public health stakeholders should continue with the awareness creation campaigns to enlighten the public regarding the ineffectiveness of herbal products and traditional medicine [28]. In addition, they should emphasize the need to adopt WHO recommended practices [28]. Adopting these measures will increase the effectiveness of COVID-19 management in Saudi Arabia.

## Figures and Tables

**Table 1:** Demographic Data of the Respondents in Saudi Arabia (*n* = 5483).

Demographic Factors		Frequencies (f)	Percentage (%)
Age	18–35 years	3722	67.9%
	36–50 years	1177	21.5%
	51–65 years	544	9.9%
	Above 65 years	39	0.7%
Gender	Female	2204	40.2%
	Male	3278	59.8%
Career	Non-health professional	4415	80.5%
	Health professional	1067	19.5%
Level of education	Primary school	52	0.9%
	High school	1502	27.4%
	Intermediate school	200	3.6%
	University	3729	68.0%

**Table 2:** COVID-19 awareness among the Saudi population. (*n* = 5483).

COVID-19 Awareness		Frequency (f)	Percentage (%)
Sources of information	Social media	4189	76.4%
	TV	1049	19.1%
	Friends chat	125	2.3%
	Newspapers	120	2.2%
Incubation period	I don’t know	226	4.1%
	Less than 2 days	44	0.8%
	2–5 days	363	6.6%
	6–14 days	4850	88.5%
Vulnerable age group	Less than 15 years	115	2.1%
	Between 16 and 30	65	1.2%
	Between 31 and 50	238	4.3%
	Above 50	5065	92.4%
Treatment	I don’t know	1569	28.6%
	Antibiotics	985	18.0%
	Symptomatic therapy	1284	23.4%
	Vitamins	1645	30.0%
Hygiene	Wash hands with water and soap	4793	87.4%
	Use hand sanitizer	667	12.2%
	Use water only	23	0.4%
Causes	Immune deficiency	1434	26.2%
	Viruses	3282	59.9%
	Population crowds	695	12.7%
	Fungi	72	1.2%
COVID-19 prevention	Social distancing	3673	67.0%
	Wearing mask	1475	26.9%
	Wearing gloves	37	0.7%
	Wearing gowns	298	5.4%

## Data Availability

The data presented in this study are available on request from the corresponding author.
